# Functional data analysis of heart rate variability from continuous ECG monitoring in older adults with and without mild cognitive impairment

**DOI:** 10.3389/fnagi.2025.1707771

**Published:** 2026-01-05

**Authors:** Jiyue Qin, Carol A. Derby, Grace Liu, Cuiling Wang, Richard P. Sloan

**Affiliations:** 1 Department of Epidemiology and Population Health, Albert Einstein College of Medicine, Bronx, NY, United States; 2 Division of Biostatistics and Bioinformatics, Herbert Wertheim School of Public Health and Human Longevity Science, University of California, San Diego, La Jolla, CA, United States; 3 Saul R. Korey Department of Neurology, Albert Einstein College of Medicine, Bronx, NY, United States; 4 Division of Behavioral Medicine, Department of Psychiatry, Columbia University Irving Medical Center, New York, NY, United States; 5 New York State Psychiatric Institute, New York, NY, United States

**Keywords:** heart rate variability, cognition, aging, functional data analysis, ambulatory measures

## Abstract

**Background:**

Reduced heart rate variability (HRV) has been associated with cognitive decline in older adults. However, prior research relied on brief in-clinic electrocardiography (ECG) recordings to measure HRV. Using 7-day continuous ambulatory ECG monitoring, we investigated time-specific differences in HRV (i.e., differences in HRV at each time point over the course of a 24-h day) between individuals with mild cognitive impairment (MCI) and those who were cognitively normal (CN) in a cohort of community-dwelling older adults.

**Methods:**

Analyses included 81 dementia-free participants [mean age = 78, standard deviation (SD) = 5, age range = 72–95; 82% female; 38% non-Hispanic White individuals, 43% non-Hispanic Black individuals]. Among them, 20 met the Jak/Bondi criteria for MCI. Participants were instructed to wear a single-lead ECG monitor continuously for 7 days. Power spectral analyses were used to determine HRV in the high-frequency band (0.15–0.40 Hz, HF-HRV) over consecutive 5-min epochs throughout the recording. Functional additive mixed models were used to analyze participants’ 24-h HF-HRV profiles to examine time-specific HRV differences between MCI and CN, after adjusting for age, sex, ethnicity, and education and further adjusting for depression, history of diabetes, and hypertension.

**Results:**

Compared to the CN group, the MCI group showed reduced HRV in the early morning (before 7 a.m.) and evening (after 7 p.m.), with the greatest difference occurring around midnight (difference: 0.6, 95% pointwise CI: 0.2, 1.1, Cohen’s *d*: 0.75).

**Conclusion:**

Our findings highlight HRV’s dynamic nature and the need to consider the time of day when investigating the relationship between HRV and cognition. Compared to daytime HRV, reduced nighttime HRV may have a stronger association with worse cognition.

## Introduction

Heart rate variability (HRV), reflecting the influence of the autonomic nervous system, has emerged as a non-invasive biomarker of neurocardiac function (Shaffer and Ginsberg, 2017). Lower HRV has been associated with several risk factors for cognitive decline, including smoking, obesity, diabetes, hypertension, depression, and inflammation (Frewen et al., 2013; Zeki Al Hazzouri et al., 2014). A direct association between reduced HRV and poorer cognition has been reported in several cohorts of older adults. However, the majority of studies measured HRV from brief in-clinic ECG recordings (≤5 min) (Frewen et al., 2013; Zeki Al Hazzouri et al., 2014; Mahinrad et al., 2016; Schaich et al., 2020; Kim et al., 2006), and only a few studies used 24-h ambulatory ECG monitoring (Zulli et al., 2005; Dalise et al., 2020).

Recent technological advancements in wearable devices have enabled accurate long-term continuous ECG monitoring over multiple days, thereby offering more reliable measurements. However, the majority of studies condense these long-term recordings into a single summary measure, typically the mean or median, when examining the relationship between HRV and other variables (Dalise et al., 2020; Calvert and Wall, 2001; Bell et al., 2019; Derby et al., 2025). Although this approach reflects the overall level of an individual’s HRV and simplifies the data structure, it fails to capture the fluctuations of HRV throughout the day in participants’ natural environments and suffers loss of data richness (Hinde et al., 2021). Moreover, the results may be sensitive to the chosen summary metric and may be hard to interpret due to the involvement of intermediate summary metrics.

Functional data analysis (FDA) provides an approach to understand the time-specific association (i.e., the association at each time point over the course of a 24-h day) between HRV and cognition by directly modeling each participant’s 24-h HRV profile (i.e., HRV at each time point over 24 h). Using this approach, both HRV and its relationship to cognition are treated as functions of the time of day and thus are allowed to vary flexibly over 24 h. The approach may elucidate how HRV’s relationship with cognition changes throughout a day and shed light on the underlying biological mechanisms.

The present study aimed to examine the time-specific differences in HRV, derived from 7-day continuous ambulatory ECG monitoring, between people with mild cognitive impairment (MCI) and those who were cognitively normal (CN), in an ethnically diverse cohort of community-dwelling older adults from the Einstein Aging Study (EAS). We hypothesized that, compared to people who were in the CN group, people with MCI would have lower HRV and that the magnitude of this difference would vary over the course of a 24-h day.

We focused on high-frequency HRV (0.15–0.40 Hz, HF-HRV) because it specifically indexes cardiac vagal control and has been linked with cognitive impairment (Shaffer and Ginsberg, 2017; Kim et al., 2006). Other HRV indices, such as low-frequency HRV (0.04–0.15 Hz, LF-HRV) and SD of normal-to-normal (SDNN) intervals, reflect the joint effects of the sympathetic and parasympathetic nervous systems (Shaffer and Ginsberg, 2017), making them difficult to interpret since they represent HRV derived from multiple sources. We did not consider indices such as root mean square of successive differences (RMSSDs) or the percentage of adjacent NN intervals that differ from each other by more than 50 ms (pNN50) because they are highly correlated with HF-HRV and would be expected to reflect similar parasympathetic modulation (Shaffer and Ginsberg, 2017).

## Methods

### Study population

We used data from the EAS, a prospective cohort study of community-dwelling older adults in Bronx County, NY, U.S. (Katz et al., 2012). Participants were recruited by systematic sampling of New York City Registered Voter Lists for Bronx County. The exclusion criteria included age <70 years, significant hearing or vision loss, current substance abuse, severe psychiatric symptoms that may interfere with testing, chronic medicinal use of opioids or glucocorticoids, cancer treatment within the last 12 months, and a diagnosis of dementia at enrollment. Participants provided written informed consent, and all procedures were approved by the Institutional Review Board of the Albert Einstein College of Medicine.

**Figure 1:**
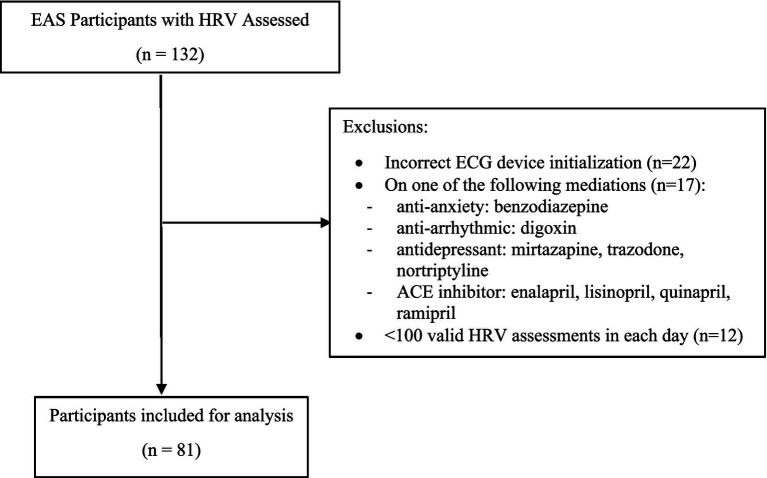
Sample selection flowchart.

Between February 2018 and March 2020, 132 dementia-free EAS participants successfully completed an ambulatory HRV protocol. Participants were excluded from the analysis if they (1) did not have correct ECG device initialization to set the clock (*N* = 22), (2) reported using medications known to affect the autonomic nervous system (mirtazapine, trazodone, digoxin, angiotensin-converting enzyme (ACE) inhibitors, benzodiazepines, and nortriptyline; *N* = 17), or (3) had <100 valid 5-min HRV epochs identified by the HRV algorithm within each day of the recording (*N* = 12, see details in the subsection “HRV Assessments”). Of note, the first exclusion criterion was necessary to ensure the accuracy of timestamps in the ECG recordings. The final analytic sample comprised 81 individuals (Figure 1).

### HRV assessments

Participants were fitted with a single-lead ECG device (MOX3 ECG and physical activity recording system; Maastricht Instruments, Maastricht, NL) at an in-person study clinic visit. They were instructed to wear the device continuously for 7 days. Participants returned devices at their second visit, which was part of the overall EAS annual protocol. The continuous ECG data were downloaded from the device and submitted to an R-wave detection routine implemented by custom-written software, producing an RR interval series. Errors in marking R waves due to excessive signal noise or ectopic events were corrected by interpolation when possible; otherwise, they were excluded from further analysis.

High-frequency (0.15–0.40 Hz) HRV, in units of ms^2^, was computed based on 5-min epochs using an interval method for computing Fourier transforms similar to that described by DeBoer et al. (1984). HRV values were natural log-transformed, denoted as Ln (HF-HRV). Epochs with more than 20% RR intervals requiring interpolation were excluded from the analysis.

### Cognitive status

Participants were classified as either the MCI group or the CN group by the Jak/Bondi actuarial criteria (Jak et al., 2009; Bondi et al., 2014). The classification used the following 10 neuropsychological instruments, with two in each of the five cognitive domains (Katz et al., 2021): (1) memory: free recall from the Free and Cued Selective Reminding Test (Buschke, 1984) and Benson Complex Figure Test [delayed (Possin et al., 2011)]; (2) executive function: Trail Making Test Part B [limit time: 300 s (Reitan, 1958)], Phonemic Verbal Fluency Test [Letters F and L for 1 min each (Benton et al., 1994)]; (3) attention: Trail Making Test Part A [limit: 150 s (Reitan, 1958)], number span [forward and backward (Weshsler, 1997)]; (4) language: Multilingual Naming Test [MINT, total score (Stasenko et al., 2019)], Category Fluency Test [animals and vegetables: 1 min each (Monsch et al., 1992)]; and (5) visual–spatial: Benson Complex Figure Test [immediate (Possin et al., 2011)] and WAIS III Block Design (Weshsler, 1997). The following actuarial formula was used: (1) impaired scores, defined as more than 1 SD below the age-, sex-, and education-adjusted normative means, on both measures within at least one cognitive domain (i.e., memory, language, or speed/executive function); (2) one impaired score, defined as more than 1 SD below the age, sex, and education adjusted normative mean, in each of three of the five cognitive domains measured; or (3) a score of 4 on the Lawton–Brody scale, indicating dependency on all four instrumental activities items (Lawton and Brody, 1969). Otherwise, an individual was classified as being in the CN group.

### Covariates

The following covariates were considered: age, sex, self-reported ethnicity, years of education, history of myocardial infarction, stroke, hypertension, diabetes (each defined as a self-report of having ever been diagnosed by a physician), and depression [defined as a score of >5 on the 15-item Geriatric Depression Scale (Yesavage and Sheikh, 1986)].

### Statistical analysis

Participant characteristics were summarized and compared by cognitive status (MCI vs. CN) using Wilcoxon rank sum tests for continuous variables and Pearson’s chi-squared tests or Fisher’s exact tests for categorical variables.

We then applied the functional additive mixed model proposed by Scheipl et al. (2015) to analyze the 24-h HF-HRV profile (natural log-transformed for normality) of each subject, denoted as $Y_{i}(t)$ , where i represents the index of the subject and t represents the time of day. Following Goldsmith et al.’s (2024) study, for a given person, the observed $Y_{i}(t)$ was obtained by averaging HF-HRV at each time point separately across the days. In this data aggregation step, days with <100 valid HRV assessments were considered invalid and excluded. Focusing on days with sufficient data is a common approach for handling missing data in wearable device studies (Di et al., 2022). However, we acknowledge the arbitrariness in the “data sufficiency” threshold and thus performed sensitivity analyses after discarding days with <200 HRV assessments. The primary model included covariates of age, sex, ethnicity, and education and can be written as:


$$\begin{array}{c} Y_{i}(t)=\beta_{0}(t)+\beta_{1}(t){\mathrm{MCI}}_{i}+\beta_{2}(t){\mathrm{Age}}_{i}+\beta_{3}(t){\text{Female}}_{i}\\ +\beta_{4}(t){\text{BlackEthnicity}}_{i}+\beta_{5}(t){\text{OtherEthnicity}}_{i}\\ +\beta_{6}(t){\text{Education}}_{i}+b_{i}+\epsilon_{i}(t), \end{array}$$

where ${\mathrm{Age}}_{i}$ and ${\text{Education}}_{i}$ are the age and education in years centered at sample average, $\beta_{0}(t)$ is the functional intercept and can be interpreted as the expected HF-HRV profile for the reference group (i.e., a typical white cognitively normal male of average age and years of education), and $\beta_{1}(t),\beta_{2}(t),\ldots ,\beta_{6}(t)$ are the functional fixed effects of each corresponding predictor. In contrast to parametric approaches where the function form is specified, here, $\beta_{0}(t),\beta_{1}(t),\ldots ,\beta_{6}(t)$ are all assumed to be smooth but unknown functions over t , which allows the intercept and fixed effects to vary flexibly over t . $b_{i}$ is a scalar subject-specific random intercept, which follows Gaussian distribution with mean zero. $\epsilon_{i}(t)$ is a Gaussian white-noise error process that consists of independent and identically distributed (iid) Gaussian variables with mean zero and constant variance. In the secondary model, we further controlled for history of diabetes and history of hypertension. Histories of myocardial infarction and stroke were not controlled due to their low prevalence in this sample.

All statistical analyses were performed using R version 4.1.2 (R Core Team, 2021). The functional additive mixed model was fitted by the pffr() function in the R package “refund” with restricted maximum likelihood (REML) estimation (Goldsmith et al., 2024). The codes for model specifications are provided in the Supplementary file.

## Results

The final analytic sample consisted of 81 participants. The average age of the participants was 78.3 (SD = 5.2) years old and the average years of education was 14.9 (SD = 3) (Table 1). Female individuals comprised 81.5% of the sample. The sample was racially and ethnically diverse (38.3% of non-Hispanic White people, 43.2% non-Hispanic Black people, and 18.5% other ethnicities). In our sample, 18 and 68% of the participants had a self-reported history of diabetes and hypertension, respectively. On average, participants wore the ECG recording device for 7.7 days and had 6.6 valid ECG recording days—days with at least 100 5-min HRV assessments were considered valid. The person-level mean Ln (HF-HRV) was 4.5 ± 0.8 ln ms^2^. In this study, the person-level mean Ln (HF-HRV) is defined as the average Ln (HF-HRV) across all the HRV assessments within a person, which is a common summary measure to reflect the overall HRV level in traditional analyses.

**Table 1:** Participant characteristics in the full sample, stratified by cognitive status.

Characteristics	Analysis full sample (*N* = 81)	Cognitively normal (CN, *N* = 61)	Mild cognitive impairment (MCI, *N* = 20)	*p*-value (MCI vs. CN)	EAS cohort at baseline (*N* = 449)
Age				0.132	
Mean (SD)	78.3 (5.2)	78.9 (5.6)	76.4 (3.4)		78.3 (5.3)
Median (IQR)	77.4 (74.1, 80.5)	77.7 (74.2, 80.7)	75.4 (73.8, 78.9)		77.3 (74, 82.2)
Years of education				0.868	
Mean (SD)	14.9 (3.3)	14.9 (3.1)	14.7 (4)		15 (3.5)
Median(IQR)	15 (12, 18)	15 (12, 18)	15 (13.8, 16.5)		15 (12, 18)
Sex, *N* (%)				0.751	
Male	15 (18.5)	12 (19.7)	3 (15)		154 (34.3)
Female	66 (81.5)	49 (80.3)	17 (85)		295 (65.7)
Ethnicity, *N* (%)				0.044	
Non-Hispanic White people	31 (38.3)	27 (44.3)	4 (20)		209 (46.5)
Non-Hispanic Black people	35 (43.2)	26 (42.6)	9 (45)		167 (37.2)
Other ethnicities	15 (18.5)	8 (13.1)	7 (35)		73 (16.3)
History of diabetes, *N* (%)	15 (18.5)	12 (19.7)	3 (15)	0.751	110 (24.5)
History of hypertension, *N* (%)	55 (67.9)	41 (67.2)	14 (70)	1	311 (69.3)
History of myocardial infarction, *N* (%)	3 (3.7)	2 (3.3)	1 (5)	1	33 (7.3)
History of stroke, *N* (%)	3 (3.7)	3 (4.9)	0 (0)	0.571	41 (9.1)
Number of total ECG recording days				0.482	
Mean (SD)	7.7 (1)	7.6 (1.1)	7.8 (0.7)		
Median (IQR)	8 (8, 8)	8 (7, 8)	8 (8, 8)		
Range	(2, 10)	(2, 10)	(5, 8)		
Number of valid ECG recording days^a^				0.977	
Mean (SD)	6.6 (1.5)	6.6 (1.5)	6.6 (1.5)		
Median (IQR)	7 (6, 8)	7 (6, 8)	7 (6, 8)		
Range	(2, 9)	(2, 9)	(2, 8)		
Person-level mean Ln (HF-HRV) (unit: ln ms^2^)^b^				0.049	
Mean (SD)	4.5 (0.8)	4.6 (0.9)	4.2 (0.6)		
Median (IQR)	4.5 (4, 5)	4.6 (4.1, 5.2)	4.3 (3.8, 4.7)		
Range	(2.8, 6.8)	(2.8, 6.8)	(3.1, 5)		

a Days with at least 100 HRV assessments were considered valid days and included in the subsequent analyses.

b Person-level mean Ln (HF-HRV) was calculated as the average Ln (HF-HRV) across all the HRV assessments within a person.

In total, 20 participants (25%) were classified as the MCI group (Table 1). Compared to the CN group, the MCI group had a smaller percentage of non-Hispanic White individuals (20% vs. 44%), a larger percentage of people belonging to other ethnicities (35% vs. 13%), and a smaller person-level mean Ln (HF-HRV) (4.2 vs. 4.6 ln ms^2^). All other characteristics were similar between the CN group and the MCI group. This analytic sample was similar to the overall EAS cohort for all characteristics except for sex, where the analytic sample had a higher percentage of female participants (84.4% vs. 65.7%).

**Figure 2:**
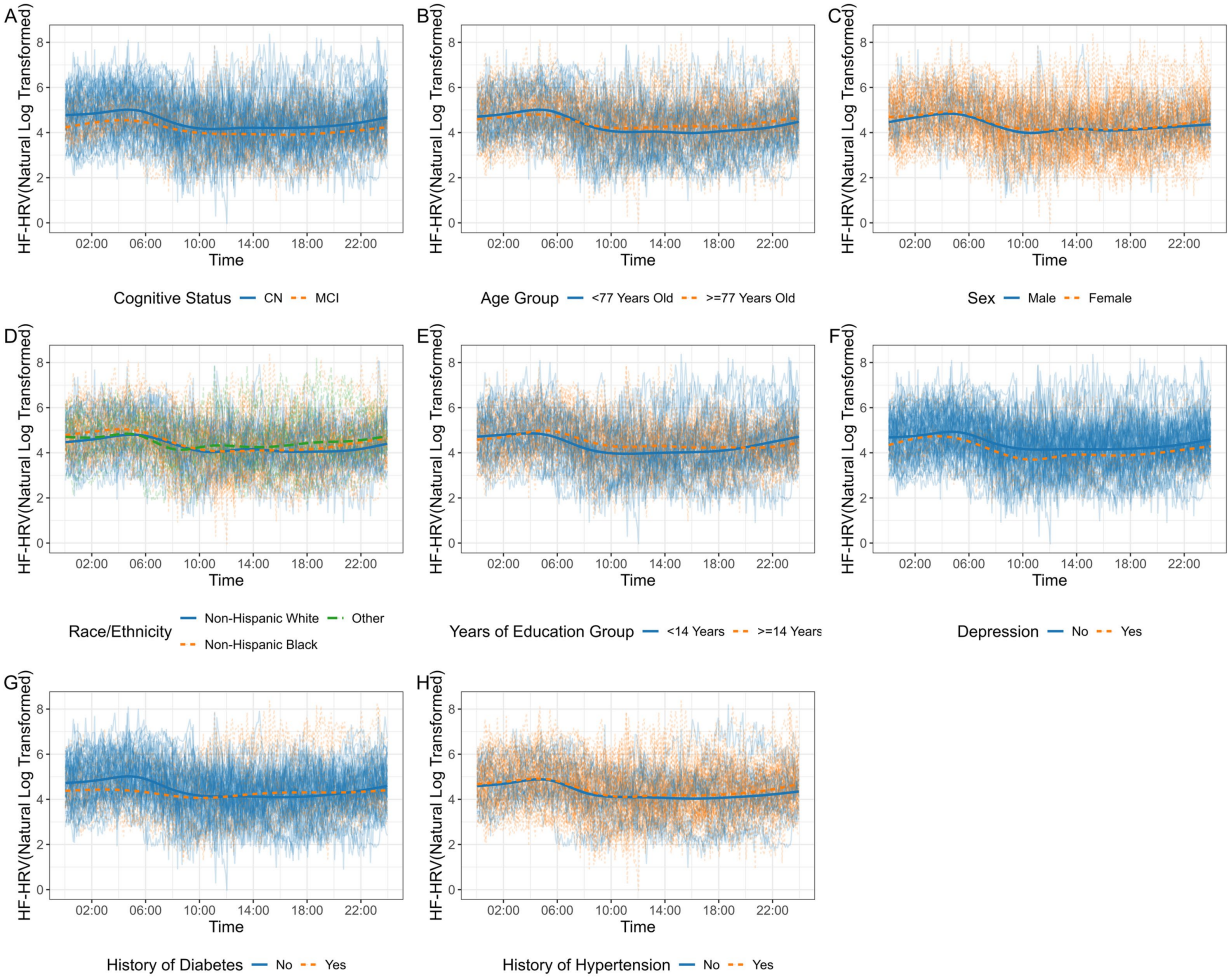
Observed 24-h HF-HRV profiles for all participants (faded curves) and group averages (bold curves), colored by group. **(A)** Cognitive status. **(B)** Age group. **(C)** Sex. **(D)** Race/ethnicity. **(E)** Years of education. **(F)** Depression. **(G)** History of diabetes. **(H)** History of hypertension. HF-HRV was natural log-transformed. Continuous variables of age and years of education were dichotomized at the sample median. CN, cognitively normal; MCI, mild cognitive impairment.

To visualize the 24-h HF-HRV profiles and how they differ between the groups, we plotted the observed 24-h HF-HRV profiles for all participants and group averages (i.e., the average HF-HRV at each time point across all participants within a group) in Figure 2. For cognitive status, the average HF-HRV was lower in the MCI group than in the CN group at all time points over 24 h. The magnitude of the difference appeared to be greater at approximately 6 a.m. and around midnight. For the other variables of age, sex, ethnicity, and education, no obvious group differences were observed from the plots.

**Figure 3:**
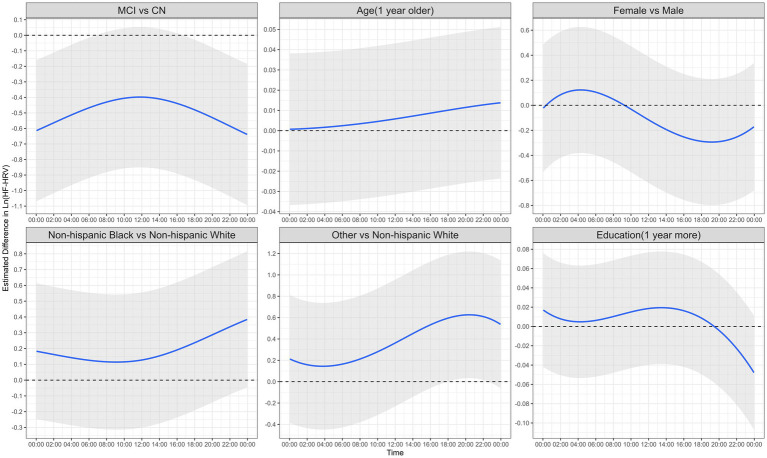
The functional additive mixed model results of 24-h HF-HRV profiles (*n* = 81, days with at least 100 HRV assessments were considered valid days and included in the analyses). The solid blue line is the estimated effect of each predictor on the outcome of HF-HRV, after adjusting for all other predictors in the figure, with shading showing the 95% pointwise confidence interval. The dotted black line is the horizontal line of zero; if it is not completely covered by the shading, the predictor is significantly associated with HF-HRV for at least one time point (i.e., MCI vs. CN, other races vs. non-Hispanic White people); otherwise, it is not significantly associated (i.e., age, female vs. male, non-Hispanic Black individuals vs. non-Hispanic White individuals, and education). Ln (HF-HRV), natural log-transformed HF-HRV; MCI, mild cognitive impairment; CN, cognitively normal.

To formally test the association between cognitive status and 24-h HF-HRV profiles, after adjusting for covariates of age, sex, ethnicity, and education, we applied the functional additive mixed model. Figure 3 shows the estimates of the fixed effect of each predictor (solid blue line) with their pointwise 95% confidence intervals (shading). For MCI, in the early morning (before 7 a.m.) and evening (after 7 p.m.), the shading was completely below the horizontal line of zero, indicating that, during these time periods, people with MCI had significantly lower HF-HRV than people who were in the CN group. The curve of the estimate also suggests that the group difference in HF-HRV between MCI and CN was the largest around midnight, with a difference of approximately 0.6 ln ms^2^ (Cohen’s *d*: 0.6/0.8 = 0.75, 95% pointwise CI: 0.2, 1.1). Compared to individuals in the CN group, those in the MCI group had a 0.6-point decrease (corresponding to a 0.75 SD decrease) in HF-HRV (natural log-transformed) around midnight. The group difference was not significant from 7 a.m. to 7 p.m. The results remained very similar after further adjusting for depression, history of diabetes, and hypertension (Supplementary Figure 1). All analyses were repeated by excluding days with <200 HRV assessments, and the results remained similar (Supplementary Figures 2, 3).

Since MCI reflects impairment across multiple cognitive domains, we further investigated the relationships between HF-HRV and impairment in five specific domains: memory, attention, executive function, language, and visuospatial ability. As shown in Supplementary Figure 4, the time-specific association between MCI and HF-HRV was also evident for the memory domain but not for the other cognitive domains.

## Discussion

In this study, we performed functional data analysis to evaluate the time-specific difference in HF-HRV, derived from 7-day continuous ambulatory ECG monitoring, between individuals with MCI and those who were in the CN group in a racially and ethnically diverse cohort of community-dwelling older adults from the EAS. We found that individuals in the MCI group had reduced HRV in the early morning (before 7 a.m.) and evening (after 7 p.m.), after adjusting for age, sex, ethnicity, and education and further adjusting for a history of diabetes and hypertension. The group difference varied over time and was the greatest around midnight (difference: 0.6, 95% pointwise CI: 0.2, 1.1, Cohen’s *d*: 0.75). Our study highlights HRV’s dynamic nature and the importance of accounting for the time of day in evaluating HRV’s relationship to cognition.

Previous research linking HRV and cognition has yielded mixed results. A study of 4,763 Irish participants (mean age = 62) showed a cross-sectional association between reduced HRV (metrics: SD of normal-to-normal (SDNN) intervals, low-frequency HRV (LF-HRV), and LF:HF ratio, derived from 5-min ECG) and poorer global cognition. However, the study reported no significant association between HF-HRV and cognition (Frewen et al., 2013). The results from a cohort of 3,583 people from Scotland, Ireland, and the Netherlands (mean age = 75) showed cross-sectional and longitudinal associations between reduced HRV (metric: SDNN, derived from 10-s ECG) and poorer performance in processing speed (Mahinrad et al., 2016). However, a study of 5,375 UK participants (mean age = 58) reported no consistent cross-sectional or longitudinal association between HRV (metrics: SDNN, LF-HRV, and HF-HRV, derived from 5-min ECG) and global cognition (Britton et al., 2008). Another study of 80 people (age ≥ 65) reported no HRV (metrics: LF-HRV, HF-HRV, and LF:HF ratio, derived from 5-min ECG) difference between participants in the MCI group and those in the CN group (Nicolini et al., 2014). Among the few studies using ambulatory ECG monitoring, a study of 311 women (aged 65–85 years) from Baltimore revealed a cross-sectional association between reduced HRV (metrics: HF-HRV, root mean square of successive differences (RMSSDs), and the number of interval differences of successive normal RR intervals greater than 50 ms (NN50), derived from a 2-h ambulatory ECG recording) and a higher risk of cognitive impairment (Kim et al., 2006). However, a study of 117 people (mean age = 74) reported no cross-sectional association between global cognition and HF-HRV derived from 24-h ECG monitoring (Dalise et al., 2020). Similarly, another study of 39 participants with MCI and 29 cognitively healthy controls reported no group difference in HF-HRV derived from 24-h ECG monitoring (Zulli et al., 2005). This inconsistency in results may be due to the failure to account for the time of day by either using a brief ECG to measure HRV or reducing the HRV data from ambulatory ECG monitoring to a single summary measure in the analyses.

Our analysis demonstrates the time-specific pattern in the HF-HRV difference between the MCI group and the CN group. We found a significant difference in the early morning (before 7 a.m.) and evening (after 7 p.m.), which is in line with one study on nighttime HF-HRV (Kong et al., 2021). This could be due to the fact that this time period corresponds to the sleep time, when fewer external stimuli are present and participants are primarily in a lying position. Compared to the daytime, nighttime may have fewer confounding factors that can influence HRV such as stressful events, physical movement, and change of positions (Shaffer and Ginsberg, 2017), thus providing a cleaner comparison between MCI and CN and, in turn, leading to a more prominent difference in HRV. Another possibility is that, during the day, cardiac autonomic function is largely the product of the parasympathetic nervous system (PNS) and the sympathetic nervous system (SNS) (Ewing et al., 1985). If the effect of the autonomic nervous system on cognitive function is largely related to the PNS, the MCI-CN difference in HF-HRV may be more evident at nighttime due to a reduced contribution from the SNS.

Several mechanisms may explain the difference in HRV between the MCI and the CN group. First, reduced HRV (RMSSD, derived from 24-h ECG monitoring) has been associated with white matter lesions in patients with MCI (Galluzzi et al., 2009). Second, inflammatory activity has been negatively associated with cognition (Schram et al., 2007; van Oijen et al., 2005; Tampubolon, 2016), and inflammatory markers, including interleukin-6, white blood cell count, fibrinogen, and C-reactive protein, are inversely related to HF-HRV (Williams et al., 2019). Therefore, a positive relationship between HF-HRV and cognitive function is consistent with the cholinergic anti-inflammatory pathway (Tracey, 2002). Finally, neurodegenerative changes in the brain during cognitive decline may alter autonomic pathways and lead to autonomic dysfunction (Mielke et al., 2012). Since these associations were based on cross-sectional analyses, no causal attributions were possible.

Our study has several strengths. First, our sample was ethnically diverse and population-based. Second, our use of ambulatory 24-h ECG monitoring over multiple days enhanced measurement reliability and provided HRV measurements in subject’s real-life settings. Moreover, the application of functional data analysis accounted for the dynamic nature of HRV and allowed the investigation of the diurnal pattern in MCI’s association with HRV. Compared to the traditional approach using summary measures, this approach leverages the richness of data, provides clearer interpretations by avoiding intermediate summary values, and offers insights into the role of timing in the MCI–HRV relationship.

Our study has the following limitations. The analysis was cross-sectional; therefore, we could not establish the temporality in the relationship between HRV and MCI. Future analyses will examine how the baseline 24-h HRV profile predicts incident MCI. Another limitation of the study is the relatively small sample size. Future studies should confirm the results in a larger sample while accounting for potential confounders and the variability in MCI severity comprehensively.

## Data Availability

The raw data supporting the conclusions of this article will be made available by the authors, without undue reservation.
