# Supplementary material for: Functional data analysis of heart rate variability from continuous ECG monitoring in older adults with and without mild cognitive impairment
Source: Front Aging Neurosci. 2026 Jan 5;17:1707771. doi: 10.3389/fnagi.2025.1707771 (PMC12813037; doi:10.3389/fnagi.2025.1707771)
Supplement: Supplementary file 1 [file Supplementary_file_1.docx]

Supplementary File


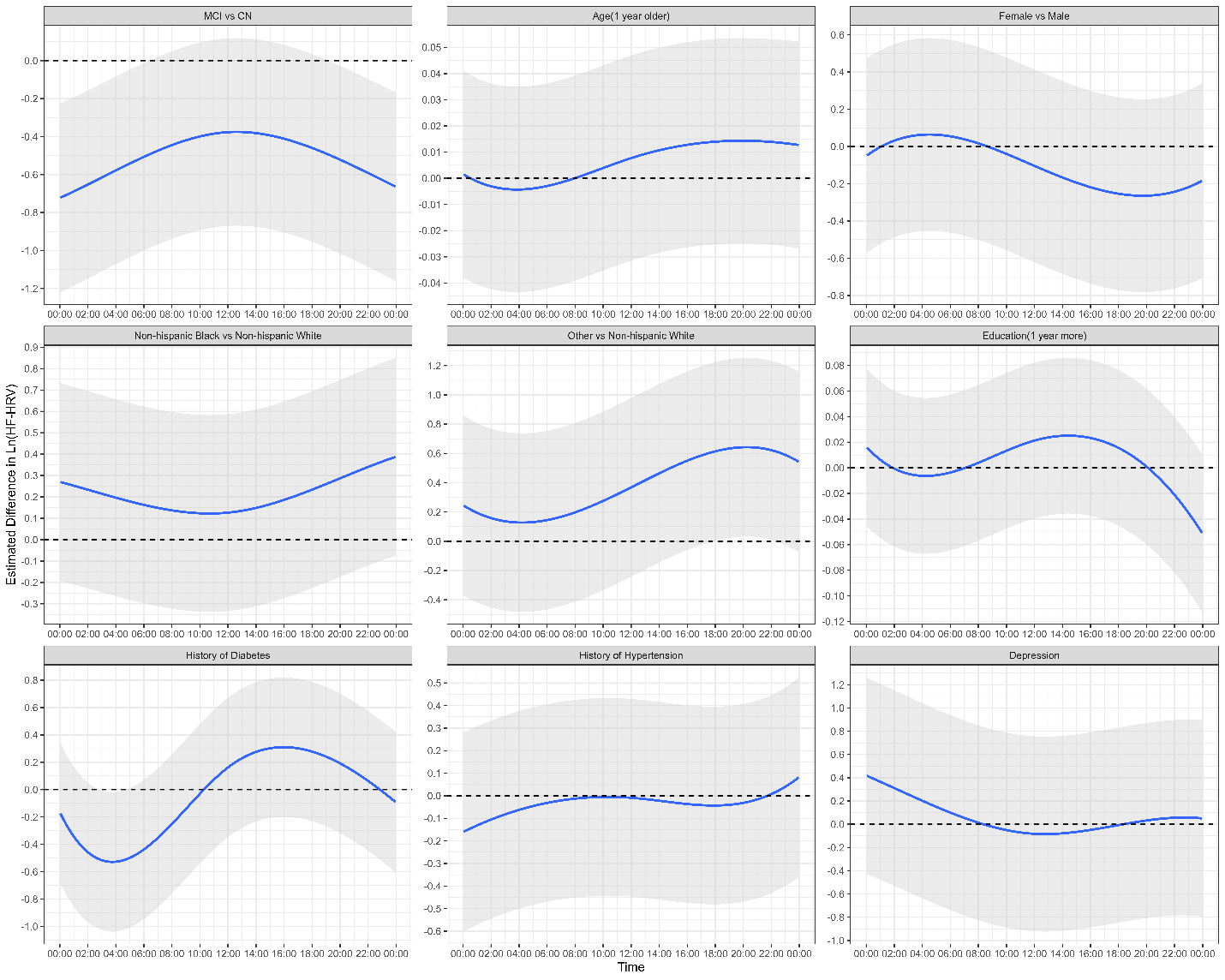


Supplementary Figure 1. The functional additive mixed model results of 24-hour HF-HRV profiles (n=81, days with at least 100 HRV assessments were considered valid days and included in the analyses). The solid blue line is the estimated effect of each predictor, controlling for all other predictors in the figure, with shading showing the 95% pointwise confidence interval. The dotted black line is the horizonal line of zero; if it is not completely covered by the shading, the predictor is significantly associated with HF-HRV for at least one timepoint (i.e., MCI vs CN, Other vs Non-Hispanic White, History of Diabetes); otherwise, it is not significantly associated (i.e., Age, Female vs Male, Non-Hispanic Black vs Non-Hispanic White, Education, History of Hypertension). Ln(HF-HRV) = natural log transformed HF-HRV; MCI = Mild Cognitive Impairment; CN = Cognitively Normal.





Supplementary Figure 2. Sensitivity analysis: The functional additive mixed model results of 24-hour HF-HRV profiles (n=68, days with at least 200 HRV assessments were considered valid days and included in the analyses). The result for MCI vs CN remained very similar. Ln(HF-HRV) = natural log transformed HF-HRV; MCI = Mild Cognitive Impairment; CN = Cognitively Normal.


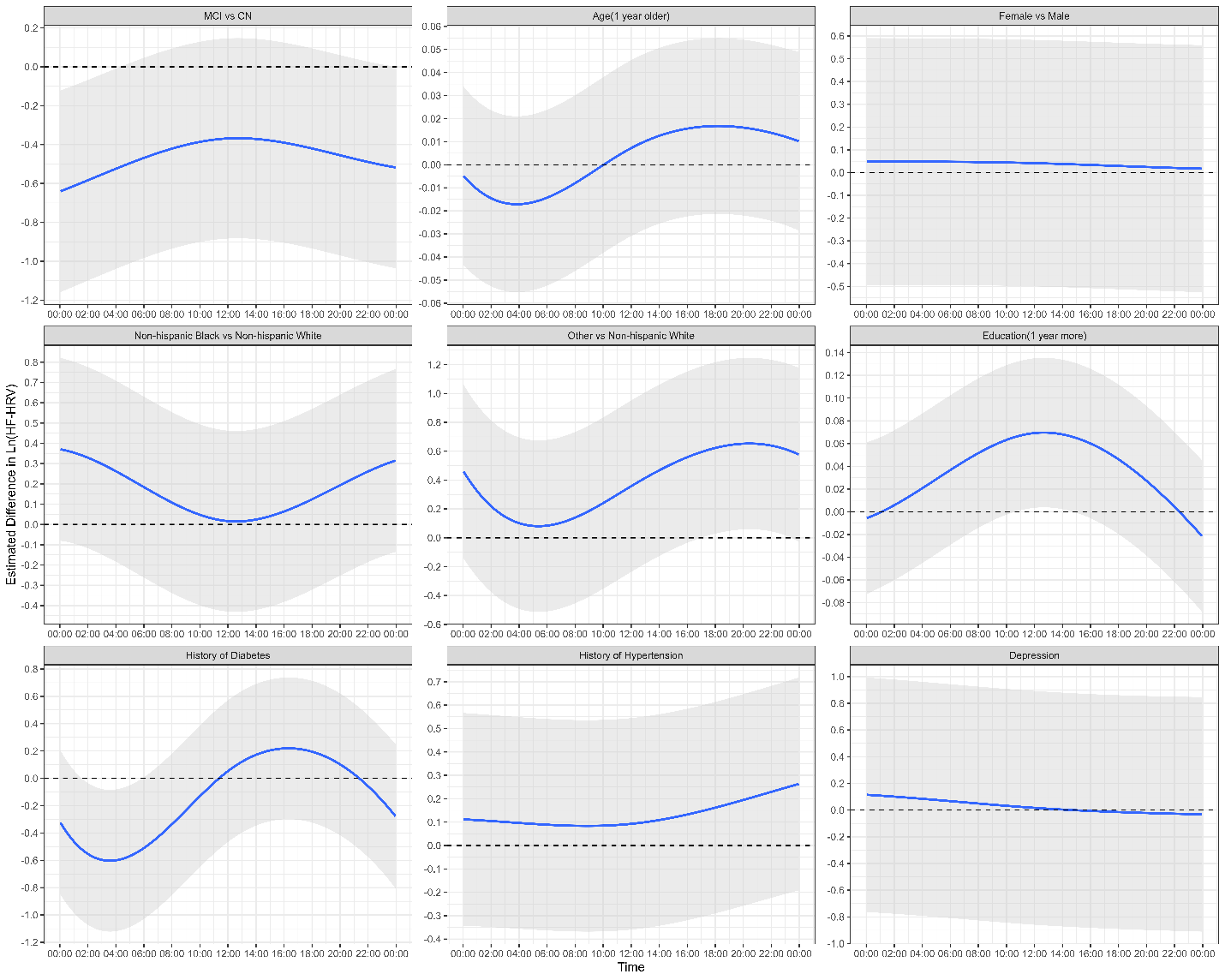


Supplementary Figure 3. Sensitivity analysis: The functional additive mixed model results of 24-hour HF-HRV profiles further adjusting for history of diabetes, history of hypertension and depression (n=68, days with at least 200 HRV assessments were considered valid days and included in the analyses). The result for MCI vs CN remained very similar. Ln(HF-HRV) = natural log transformed HF-HRV; MCI = Mild Cognitive Impairment; CN = Cognitively Normal.


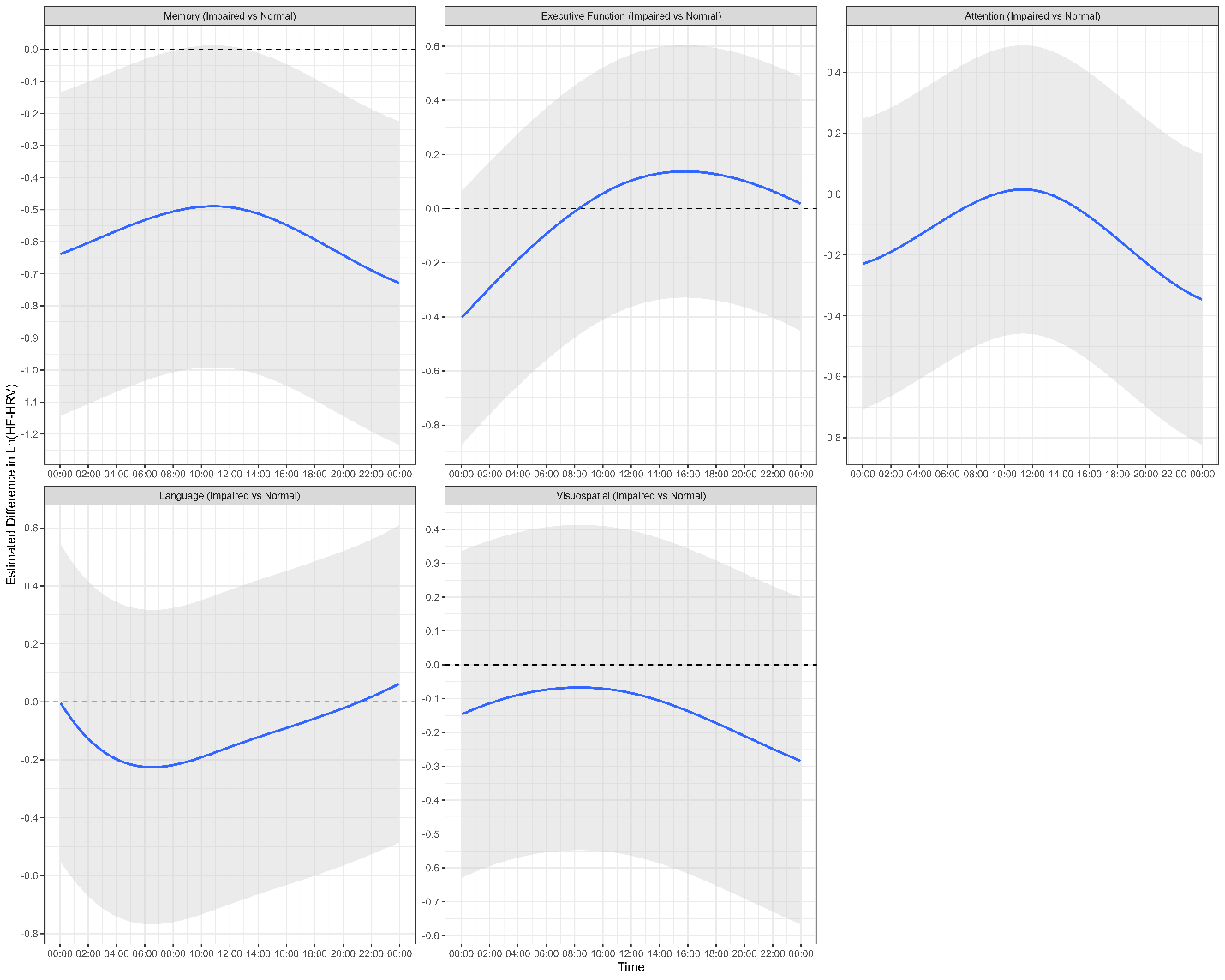


Supplementary Figure 4. The functional additive mixed model results of 24-hour HF-HRV profiles for each of the five cognitive domains. All the five models controlled for age, sex, race/ethnicity, education, depression, history of diabetes and history of hypertension. (n=81, days with at least 100 HRV assessments were considered valid days and included in the analyses). Impairment in the memory domain was significantly associated with a smaller HF-HRV.

Codes

library(refund)

# the outcome Y is 24-hour Ln(HF-HRV) profiles, which is a matrix of 81(number of subjects) * 288(288 HRV 5-minitue epochs over the course of 24 hours)

#Model 1: control for demographics + MCI

fit1 = pffr(Y ~ mci_numeric+age_center+female_numeric+educyrs_center + black_race + other_race+ c(s(id, bs = 're')),data=hrv_fda)

#Model 2: control for demographics + MCI + history of diabetes +history of hypertension+depression

fit2= pffr(Y ~ mci_numeric+age_center+female_numeric+educyrs_center + black_race + other_race+ cumul_diab + cumul_hyper +depression+ c(s(id, bs = 're')),data=hrv_fda)
